# Detecting Redundant Health Survey Questions by Using Language-Agnostic Bidirectional Encoder Representations From Transformers Sentence Embedding: Algorithm Development Study

**DOI:** 10.2196/71687

**Published:** 2025-06-10

**Authors:** Sunghoon Kang, Hyewon Park, Ricky Taira, Hyeoneui Kim

**Affiliations:** 1 College of Nursing, Seoul National University, 103 Daehak-ro, Jongno-gu, Seoul, 03080, Republic of Korea, 82 027408483; 2 The Department of Radiological Sciences, David Geffen School of Medicine, University of California, Los Angeles, Los Angeles, CA, United States

**Keywords:** person-generated health data, PGHD, bidirectional encoder representations from transformers, BERT, semantic similarity, language-agnostic BERT sentence embedding, LaBSE, sentence-bidirectional encoder representations from transformers, SBERT, interoperability

## Abstract

**Background:**

As the importance of person-generated health data (PGHD) in health care and research has increased, efforts have been made to standardize survey-based PGHD to improve its usability and interoperability. Standardization efforts such as the Patient-Reported Outcomes Measurement Information System (PROMIS) and the National Institutes of Health (NIH) Common Data Elements (CDE) repository provide effective tools for managing and unifying health survey questions. However, previous methods using ontology-mediated annotation are not only labor-intensive and difficult to scale but also challenging for identifying semantic redundancies in survey questions, especially across multiple languages.

**Objective:**

The goal of this work was to compute the semantic similarity among publicly available health survey questions to facilitate the standardization of survey-based PGHD.

**Methods:**

We compiled various health survey questions authored in both English and Korean from the NIH CDE repository, PROMIS, Korean public health agencies, and academic publications. Questions were drawn from various health lifelog domains. A randomized question pairing scheme was used to generate a semantic text similarity dataset consisting of 1758 question pairs. The similarity scores between each question pair were assigned by 2 human experts. The tagged dataset was then used to build 4 classifiers featuring bag-of-words, sentence-bidirectional encoder representations from transformers (SBERT) with bidirectional encoder representations from transformers (BERT)–based embeddings, SBERT with language-agnostic BERT sentence embedding (LaBSE), and GPT-4o. The algorithms were evaluated using traditional contingency statistics.

**Results:**

Among the 3 algorithms, SBERT-LaBSE demonstrated the highest performance in assessing the question similarity across both languages, achieving area under the receiver operating characteristic and precision-recall curves of >0.99. Additionally, SBERT-LaBSE proved effective in identifying cross-lingual semantic similarities. The SBERT-LaBSE algorithm excelled at aligning semantically equivalent sentences across both languages but encountered challenges in capturing subtle nuances and maintaining computational efficiency. Future research should focus on testing with larger multilingual datasets and on calibrating and normalizing scores across the health lifelog domains to improve consistency.

**Conclusions:**

This study introduces the SBERT-LaBSE algorithm for calculating the semantic similarity across 2 languages, showing that it outperforms BERT-based models, the GPT-4o model, and the bag-of-words approach, highlighting its potential in improving the semantic interoperability of survey-based PGHD across language barriers.

## Introduction

Person-generated health data (PGHD) is becoming increasingly important in managing individual health. PGHD encompass health-related information that individuals create and collect outside traditional clinical environments, helping them monitor and manage their well-being [1,2]. Examples of PGHD include biometric data from wearable devices and self-reported information such as patient-reported outcomes. Since PGHD has the potential for continuously capturing health insights beyond health care settings, there is growing interest in leveraging PGHD to support clinical care [3-5]. In parallel, PGHD is increasingly explored as a resource for patient-centered outcomes research [6,7]. However, there are several challenges in the effective use of PGHD, including developing robust data management systems, ensuring data security, deploying it seamlessly into clinical workflows, and maintaining high data quality [5,7,8].

Standardizing survey-based PGHD is a critical step in enabling its broader use [9]. An important aspect of standardization is to identify redundancies in the form of semantic equivalencies. These redundancies may arise because the clarity, tone, tense, directness, and formality of the language can be phrased differently for the same purposeful inquiry depending upon the author. For example, emotional symptoms may be captured by questions such as “Do you feel like withdrawing from family or friends?” or “I don’t really want to talk to people around me.” This variation makes identifying semantically equivalent questions—and thus standardizing survey-based PGHD—a complex task. Efforts such as the Patient-Reported Outcomes Measurement Information System (PROMIS) and the National Institutes of Health (NIH) Common Data Elements (CDE) repository aim to provide standardized health survey questions. PROMIS, a consensus-based item bank designed for managing patient-reported outcomes, offers standardized measures that are applicable across various diseases and clinical settings [10-12]. These measures have helped health care providers across various clinical settings, including pain management [13], orthopedics [14], and primary care [15]; in cancer care [16]; in managing patient symptoms; in tailoring treatments; and in improving communication between patients and clinicians. The NIH CDE repository, through metadata tagging, also plays a key role in standardizing data elements, including health surveys [17,18]. Both PROMIS and the CDE repository are essential for enhancing the interoperability of health data.

In practice, the deployment of PGHD acquisition applications requires that survey questions be drawn from these established standardized resources. Data collected using questions outside of these resources still require additional efforts to achieve standardization. Although previous studies have explored ontology-mediated methods to identify semantically equivalent health questions [10,11], annotating each question with ontology concepts is labor-intensive and lacks scalability as such knowledge sources expand. As a complementary approach, deep learning and transformer-based methods have been applied to semantic textual similarity (STS) tasks in clinical texts, including radiology and pathology reports [19], clinical notes [20-22], and medical question-answer pairs [23]. A range of models has been explored, such as convolutional neural networks [19]; transformer-based architectures such as bidirectional encoder representations from transformers (BERT), robustly optimized BERT approach, and XLNet [20-22]; and the Siamese network [23]. Despite their promising performance, most of these models have been limited to single-language settings—predominantly English [20-22] or Chinese [19,23]. Consequently, cross-lingual STS remains underexplored, highlighting the need for standardization efforts that promote semantic interoperability across languages.

To address these challenges, we developed Standardized PGHD Utilization Resources and Tools (SPURT), which supports the standardization and reuse of survey-based PGHD by identifying semantically equivalent questions and facilitating the storage, retrieval, and sharing of these data. Unlike PROMIS and the NIH CDE repository, SPURT annotates and stores health survey questions in both English and Korean while also detecting semantically redundant questions. This ensures the use of consistent question formats whenever possible. Technically, assessing semantic similarity between texts is well-established and widely applied for managing text resources [24]. However, SPURT faces 2 unique challenges in its assigned task. First, it must effectively assess semantic similarities within or between 2 different languages—English and Korean. Although multilingual embeddings can be used to address this challenge [25,26], they often perform less effectively for low-resource languages such as Korean compared to high-resource languages such as English [27]. One common solution is to translate low-resource languages into high-resource ones before embedding, but this approach risks losing or distorting the original meaning [28,29]. Second, it must ensure computational efficiency for real-time semantic comparisons between questions. Calculating semantic similarity by using large language models such as BERT is computationally expensive, with a time complexity of O (N!). For example, computing the similarity of approximately 10,000 sentence pairs can take around 65 hours using a V100 graphics processing unit [30]. Given that SPURT is designed to be a real-time, reactive data processing tool, achieving reasonable response times is crucial for its functionality.

This study presents the development of a novel algorithm for detecting redundant questions, addressing the challenges outlined above. The algorithm utilizes sentence-BERT (SBERT), a variant of BERT designed for efficient sentence-level semantic similarity calculations [30] along with language-agnostic BERT sentence embedding (LaBSE) [31] to enhance multilingual capability. Sentence-BERT is a model specifically designed for calculating STS between sentences, and LaBSE is an embedding that supports efficient cross-lingual STS by mapping multilingual sentences into a shared embedding space. The SBERT-LaBSE algorithm integrates the strengths of both models and facilitates the identification of semantically equivalent questions across languages.

## Methods

### Corpus Description: The STS Dataset

An STS dataset contains text pairs along with predefined similarity scores that quantify their semantic closeness [32-36]. This study shows an STS dataset that fine-tunes pretrained language models and evaluates our algorithms’ performance in determining the semantic similarity between health-related questions.

We collected English and Korean questions from self-reported questionnaires covering 5 health lifelog domains, that is, diet, physical activity, living environment, stress management, and sleep. English questions (n=1222) were sourced from the NIH CDE repository, PROMIS, and academic publications, while Korean questions (n=963) were gathered from web-based resources provided by public health agencies and hospitals in Korea [17,37-40].

To build the STS dataset, we began by randomly selecting 5 seed questions from each of the 5 health lifelog domains in Korean, resulting in 25 seed questions. For each question, correspondingly similar questions for Korean were identified, resulting in 25 similar seed questions for each language. This correspondence of seed questions was performed to minimize the effects of semantic complexity on algorithm performance. We then randomly selected 30 comparison questions for each seed question, which yielded a total of 1500 question pairs (750 in each language).

The gold standard for semantic similarity between the question pairs was determined by 2 researchers with nursing backgrounds who independently scored the similarity of each question pair, following a standardized scoring protocol (Table 1). The agreement between the researchers, as measured by Cohen κ, varied by the health lifelog domains: 0.91 for diet, 0.72 for living environment, 0.83 for physical activity, 0.86 for sleep, and 1.0 for stress management, with an average Cohen κ of 0.86 across all the health lifelog domains.

**Table 1.** Scoring protocol for semantic similarity. The seed question was “In the past month, have you ever had chest pain when you were not performing any physical activity?”

Score	Scoring protocol	Examples
4	Minor differences in word choice from the seed question but takes the same form of response	In the past month, have you had chest pain when you were not doing physical activity?
3	Share the same key topic, although some details may be added, altered, or omitted from the seed question	Do you feel pain in your chest when you do physical activity?
2	The key topics are similar but more specific or general than that of the seed question	Has your doctor ever said that you have a heart condition and that you should only perform physical activity recommended by a doctor?
1	Does not share the core topic from the seed question or belongs to a completely different health lifelog domain	Have you done general conditioning exercises in the past 4 weeks?

Upon completion of this annotation process, we observed that the initial distribution of the similarity scores was imbalanced—skewed heavily toward lower similarity scores. Only 2.3% (7/300) of the pairs received a score of 4, and 4.9% (15/300) received a score of 3. To address this imbalance, we supplemented the dataset with an additional 117 English and 142 Korean question pairs from other sources, chosen to increase the frequency of semantically similar (ie, higher scores) samples in the evaluation STS dataset. These additions brought the final evaluation set to 820 question pairs (410 in each language) with the following distribution: 12.2% (99/810), 30.5% (247/810), 26.8% (217/810), and 30.5% (247/810) for scores 4, 3, 2, and 1, respectively.

Using a similar procedure as described above, we compiled a second English STS dataset for fine-tuning our pretrained language models. This fine-tuning dataset included 938 annotated English question pairs. The fine-tuning set had a distribution of 6.2% (58/938) scoring 4, 14% (131/938) scoring 3, 23.5% (220/938) scoring 2, and 56.4% (529/938) scoring 1.

**Figure 1.**
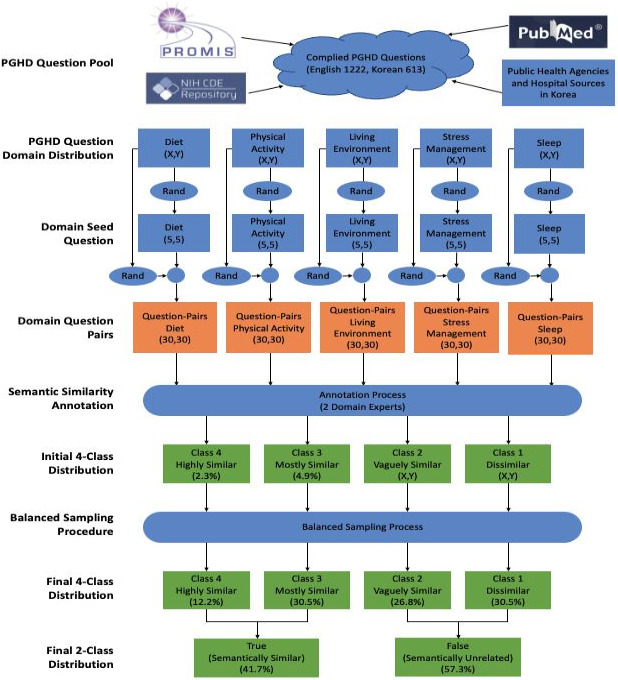
The process of preparing the semantic textual similarity dataset for fine-tuning and evaluation. CDE: common data elements; NIH: National Institutes of Health; PGHD: person-generated health data; PROMIS: Patient-Reported Outcomes Measurement Information System; Rand: random selection; STS: semantic textual similarity.

In total, the STS dataset consisted of 1758 question pairs, broken down into 820 for evaluation testing (410 English and 410 Korean) and 938 in English for classifier model refinement (see Multimedia Appendix 1). The process of constructing the STS dataset is illustrated in Figure 1.

### Semantic Similarity Calculation Algorithms

#### Overview

We developed 4 classifiers to compare their performance capability for distinguishing the binary task of semantic similarity between STS question pairs. These were (1) the bag-of-words (BoW) model, (2) SBERT with BERT-based embeddings (SBERT-BERT), (3) LaBSE, and (4) the GPT-4o model (GPT-4o). Among these, the SBERT-BERT algorithm was included to serve as a translation-dependent baseline, enabling comparison with multilingual models such as SBERT-BERT and GPT-4o. Model fine-tuning and algorithm development were performed using Python (version 3.11).

#### BoW Classifier

The BoW algorithm, a traditional language model that represents sentences by their word frequency, serves as the baseline [41]. The BoW model’s vocabulary was derived from the STS dataset, comprising 1349 unique word forms after stop-word removal and lemmatization. Each sentence was represented as a 1349D vector based on the vocabulary. Cosine similarity was used to calculate the semantic distance of the question pairs. For Korean questions, translation to English was performed using the Google Translator application programming interface prior to similarity calculation [42].

#### The SBERT-BERT Algorithm

The SBERT-BERT large language model was derived from the pretrained BERT-based model, which has 12 layers, a 768D hidden layer, 12 attention heads, and 110 million parameters [30]. SBERT-BERT supports only English. We fine-tuned the pretrained SBERT-BERT model to optimize its performance for identifying semantic equivalency among health questions by using the 938 English question pairs described above. The fine-tuning was performed with a batch size of 32, 8 epochs, and a learning rate of 2e-5, which were deemed optimal after testing various configurations. The AdamW optimizer was used for model optimization [43]. The fine-tuned SBERT-BERT algorithm was then evaluated using the test STS dataset of 410 English question pairs and 410 Korean question pairs. As previously stated, the Korean questions were translated into English using the Google Translator application programming interface to execute the evaluation.

#### The SBERT-LaBSE Algorithm

The SBERT-LaBSE algorithm differs from SBERT-BERT in that it supports multiple languages within a single embedding space [31]. The pretrained SBERT-LaBSE model was derived from the LaBSE model, which also consists of 12 layers, a 768D hidden layer, 12 attention heads, and 110 million parameters [31]. Fine-tuning was performed in the same manner as for SBERT-BERT. Unlike the other models, SBERT-LaBSE can assess the semantic similarity of English and Korean questions without requiring translation.

#### The GPT-4o Algorithm

The GPT-4o model, a state-of-the-art large language model, is designed to understand and generate text in multiple languages, including English and Korean [44]. Unlike the SBERT-BERT and SBERT-LaBSE, which rely on fixed embeddings for similarity calculation, the GPT-4o operates as a generative model that dynamically evaluates semantic similarity based on contextual understanding. However, in this study, we utilized GPT-4o in a deterministic manner to predict the score of sentence pairs. Each sentence pair was presented with a specific instruction asking to evaluate the score on a scale from 1 to 4 (Multimedia Appendix 2). Fine-tuning of the GPT-4o model was conducted using the fine-tuning application programming interface from the OpenAI platform [45].

### Performance Evaluation

The performance of the similarity calculation algorithms was evaluated as a binary classification problem to simplify interpretation. The 4-point ordinal similarity scores from the STS dataset were converted into binary labels, where scores of 3 and 4 were categorized as similar and scores of 1 and 2 as dissimilar.

**Figure 2.**
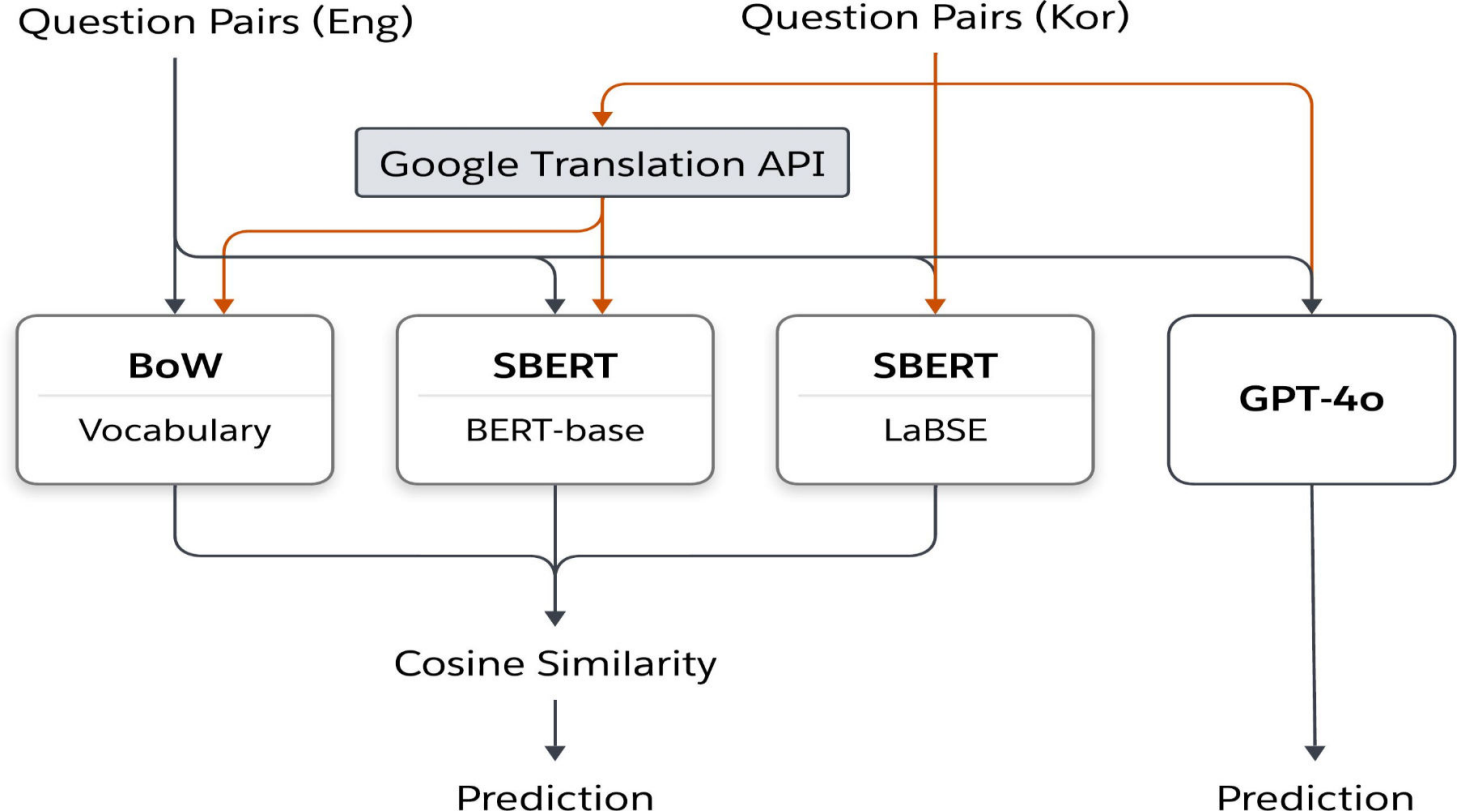
Similarity calculation with the 4 algorithms. API: application programming interface; BERT: bidirectional encoder representations from transformers; BoW: bag-of-words; Eng: English; Kor: Korean; LaBSE: language-agnostic bidirectional encoder representations from transformers sentence embedding; SBERT: sentence-bidirectional encoder representations from transformers.

Optimal thresholds for predicting similarity were determined for the continuous similarity scores, which ranged from –1 to 1. Precision, recall, and *F*_1_-scores were calculated to assess algorithm performance, and the area under the curve for both the receiver operating characteristic and precision-recall curves were examined. The processes used by the 3 algorithms to calculate similarity are illustrated in Figure 2.

### Ethical Considerations

This study does not involve human participants, intervention, or identifiable private information. The analysis was based on publicly available and nonidentifiable health survey questions from open repositories and published sources. As such, it does not fall under the scope of human subject research as defined by the Seoul National University institutional review board. According to Article 2 and Article 8, Paragraph 2 of the Seoul National University institutional review board regulations (regulation 27, effective September 11, 2023), studies that do not involve human participants or human-derived materials are exempt from institutional review board review. Therefore, this study was not submitted for ethical review. No informed consent, compensation, or privacy protection measures were applicable, as no human participants were involved, and no personal data were collected or analyzed.

## Results

**Figure 3.**
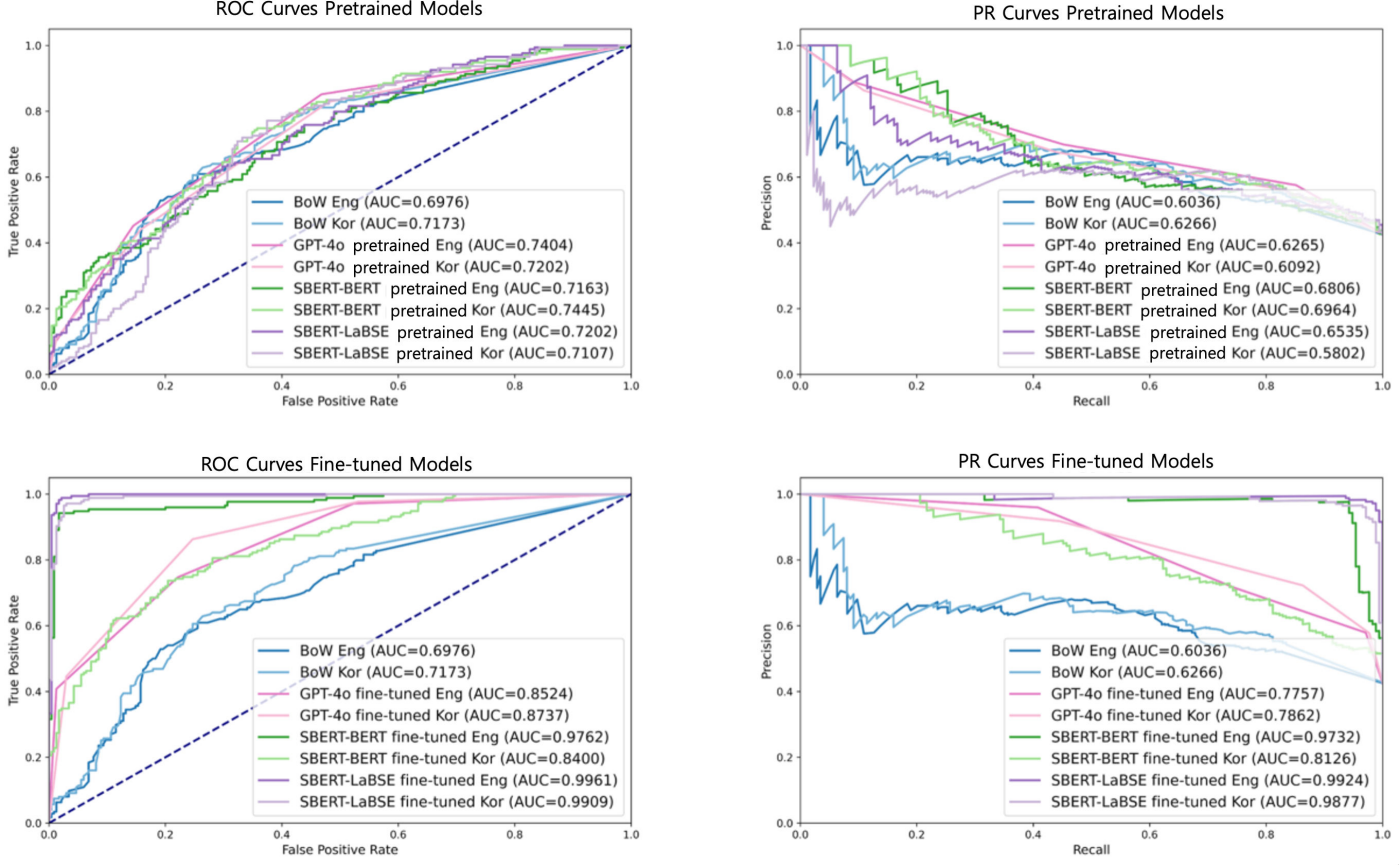
Receiver operating characteristic and precision-recall curves for pretrained and fine-tuned embeddings on English and Korean questions, combining the health lifelog domains. AUC: area under the curve; BERT: bidirectional encoder representations from transformers; BoW: bag-of-words; Eng: English; Kor: Korean; LaBSE: language-agnostic bidirectional encoder representations from transformers sentence embedding; PR: precision-recall; ROC: receiver operating characteristic; SBERT: sentence-bidirectional encoder representations from transformers.

The performance of the 3 models for classifying similar versus dissimilar question pairs when aggregating the 5 health lifelog domains is summarized in Table 2 and Figure 3. In the zero-shot trials (ie, without the model refining stage), there were minimal differences in performance among the 3 algorithms for both English and Korean questions. All algorithms exhibited higher recall than precision in both languages. After fine-tuning, the SBERT-BERT algorithm showed substantial improvement, particularly for English questions, in which the *F*_1_-score increased from 0.65 to 0.96. For Korean questions, the improvement was moderate, with the *F*_1_-score progressing from 0.68 to 0.73. In contrast, SBERT-LaBSE demonstrated significant improvements for both languages post fine-tuning. For English questions, the *F*_1_-scores increased from 0.66 to 0.98, while for Korean, the *F*_1_-scores increased from 0.68 to 0.98. Fine tuning for both SBERT-BERT and SBERT-LaBSE models resulted in noticeable balanced performance between recall and precision. Similarly, GPT-4o exhibited improved performance following fine-tuning, with its *F*_1_-scores increasing from 0.69 to 0.79 for the English questions and from 0.67 to 0.79 for the Korean questions. However, the degree of improvement was smaller than that observed in SBERT models.

**Table 2.** Performance metrics for the 3 algorithms, combining the health lifelog domains.

Performance metrics		BoW^a^	GPT-4o pretrained	GPT-4o fine-tuned	SBERT^b^ with pretrained		SBERT with fine-tuned	
					BERT-base	LaBSE^c^	BERT-base	LaBSE
English question pairs (n=410)								
Accuracy		0.6112	0.6683	0.8463	0.6308	0.5917	0.9702	0.9853
Precision		0.5279	0.5753	0.9590	0.5451	0.5111	0.9668	0.9818
Recall		0.8161	0.8514	0.6686	0.7989	0.9253	0.9632	0.9839
*F*_1_-score		0.6411	0.6866	0.7879	0.6480	0.6585	0.9649	0.9828
Korean question pairs (n=410)								
Accuracy		0.6610	0.6512	0.8488	0.6659	0.6878	0.7576	0.9839
Precision		0.5732	0.5620	0.9520	0.5760	0.6054	0.6929	0.9818
Recall		0.8057	0.8286	0.6800	0.8229	0.7714	0.7817	0.9806
*F*_1_-score		0.6698	0.6697	0.7933	0.6776	0.6784	0.7332	0.9812

a BoW: bag-of-words.

b SBERT: sentence-bidirectional encoder representations from transformers.

c LaBSE: language-agnostic bidirectional encoder representations from transformers sentence embedding.

Table 3 presents the performance of the 2 SBERT algorithms across the 5 health lifelog domains. For all the health lifelog domains, the fine-tuned SBERT-BERT and SBERT-LaBSE algorithms demonstrated high performance on English questions, with receiver operating characteristic and precision-recall area under the curve values exceeding 0.95 and approaching 0.99. However, the SBERT-BERT algorithm struggled with the English-translated Korean questions, particularly in the physical activity domain. In contrast, the SBERT-LaBSE algorithm consistently delivered strong performance across all the health lifelog domains even for Korean questions.

**Table 3.** Performance metrics of the sentence-bidirectional encoder representations from transformers–based algorithms with fine-tuned bidirectional encoder representations from transformers and language-agnostic bidirectional encoder representations from transformers sentence embedding models by the health lifelog domains.

Performance metrics		English question pairs (n=410)						Korean question pairs (n=410)					
		DL^a^ (n=80)	HLE^b^ (n=80)	PA^c^ (n=80)	Sleep (n=85)	Stress (n=85)	All	DL (n=80)	HLE (n=80)	PA (n=80)	Sleep (n=85)	Stress (n=85)	All
BoW^d^													
Accuracy		0.7215	0.7250	0.6625	0.4118	0.7176	0.6112	0.7250	0.8125	0.7250	0.5765	0.5882	0.6610
Precision		0.7000	0.6383	0.5952	0.4118	0.6279	0.5279	0.6585	0.7941	0.6275	0.0000	0.5000	0.5732
Recall		0.6176	0.8571	0.7143	1.0000	0.7714	0.8161	0.7714	0.7714	0.9143	0.0000	0.8286	0.8057
*F*_1_-score		0.6563	0.7317	0.6494	0.5833	0.6923	0.6411	0.7105	0.7826	0.7442	0.0000	0.6237	0.6698
ROC^e^ AUC^f^		0.7297	0.7457	0.6810	0.5820	0.7611	0.6976	0.7667	0.7937	0.7933	0.5937	0.6609	0.7174
PR^g^ AUC		0.7250	0.6718	0.6301	0.5025	0.6834	0.6036	0.7394	0.7373	0.7519	0.4498	0.5985	0.6267
GPT-4o fine-tuned													
Accuracy		0.9367	0.9750	0.9625	0.9765	0.9765	0.7873	0.8875	0.9375	0.7750	0.8000	0.8471	0.8293
Precision		0.8919	0.9714	0.9444	0.9459	0.9459	0.7403	0.9643	1.0000	0.8696	0.9500	0.9583	0.7586
Recall		0.9706	0.9714	0.9714	1.0000	1.0000	0.7701	0.7714	0.8571	0.5714	0.5429	0.6571	0.8800
*F*_1_-score		0.9296	0.9714	0.9577	0.9722	0.9722	0.7549	0.8571	0.9231	0.6897	0.6909	0.7797	0.8148
ROC AUC		0.9598	0.9838	0.9727	0.9863	0.9757	0.8524	0.9340	0.9444	0.8295	0.8823	0.8769	0.8737
PR AUC		0.9138	0.9672	0.9418	0.9629	0.9354	0.7757	0.8969	0.9344	0.7510	0.7969	0.8182	0.7862
SBERT^h^ with fine-tuned BERT-base^i^													
Accuracy		0.9646	0.9625	0.9800	0.9906	0.9835	0.9702	0.8525	0.8125	0.7025	0.7200	0.7882	0.7576
Precision		0.9650	0.9502	0.9784	0.9836	0.9830	0.9668	0.8037	0.7391	0.6175	0.6036	0.7108	0.6929
Recall		0.9529	0.9657	0.9771	0.9943	0.9771	0.9632	0.8800	0.8914	0.8629	0.9543	0.8229	0.7817
*F*_1_-score		0.9585	0.9571	0.9770	0.9887	0.9799	0.9649	0.8384	0.8062	0.7176	0.7376	0.7622	0.7332
ROC AUC		0.9859	0.9698	0.9923	0.9929	0.9936	0.9867	0.9125	0.8563	0.7901	0.8411	0.8462	0.8412
PR AUC		0.9858	0.9480	0.9925	0.9870	0.9918	0.9800	0.9008	0.7969	0.7640	0.8109	0.8244	0.8134
SBERT with fine-tuned LaBSE													
Accuracy		0.9848	0.9900	0.9875	0.9906	0.9906	0.9853	0.9775	0.9975	0.9850	0.9859	0.9835	0.9839
Precision		0.9716	0.9889	0.9728	0.9944	0.9889	0.9818	0.9719	0.9944	0.9836	0.9775	0.9886	0.9818
Recall		0.9941	0.9886	1.0000	0.9829	0.9886	0.9839	0.9771	1.0000	0.9829	0.9886	0.9714	0.9806
*F*_1_-score		0.9826	0.9885	0.9861	0.9884	0.9887	0.9828	0.9743	0.9972	0.9828	0.9829	0.9797	0.9812
ROC AUC		0.9965	0.9929	0.9987	0.9989	0.9979	0.9968	0.9893	0.9976	0.9962	0.9971	0.9930	0.9951
PR AUC		0.9964	0.9901	0.9984	0.9985	0.9975	0.9960	0.9872	0.9958	0.9947	0.9957	0.9927	0.9934

a DL: dietary lifestyle.

b HLE: human living environment.

c PA: physical activity.

d BoW: bag-of-words.

e ROC: receiver operating characteristic.

f AUC: area under the curve.

g PR: precision-recall.

h SBERT: sentence-bidirectional encoder representations from transformers.

i BERT: bidirectional encoder representations from transformers.

Table 4 presents the optimal cutoff values for the 3 algorithms. The pretrained SBERT-BERT and SBERT-LaBSE models showed considerable variation in the cutoff values across the 5 health lifelog domains. However, after fine-tuning, these variations decreased, indicating that fine-tuning helped stabilize the algorithms. Despite this improvement, the SBERT-LaBSE algorithm still exhibited more variability in the cutoff values across the health lifelog domains compared to SBERT-BERT, suggesting that further calibration may be required for SBERT-LaBSE. Multimedia Appendix 3 provides example question pairs from each health lifelog domain, along with the similarity scores assigned by human reviewers and predicted by the 3 algorithms.

**Table 4.** Optimal cutoff for algorithms on bag-of-words and pretrained and fine-tuned SBERT-BERT and SBERT–LaBSE in each health lifelog domain.

Health lifelog domain		Bag-of-words	SBERT^a^ with pretrained		SBERT with fine-tuned	
			BERT-base^b^	LaBSE	BERT-base	LaBSE^c^
English question pairs (n=410)						
Dietary lifestyle		0.2887	0.6274	0.5359	0.6349	0.6262
Human living environment		0.1291	0.5369	0.3965	0.6151	0.6425
Physical activity		0.3162	0.3667	0.4822	0.6304	0.6202
Sleep		0.0000	0.6790	0.2456	0.6617	0.6574
Stress		0.1054	0.5817	0.3807	0.6359	0.5958
All		0.1291	0.5816	0.3796	0.6278	0.6091
Korean question pairs (n=410)						
Dietary lifestyle		0.2887	0.5990	0.3103	0.5639	0.6568
Human living environment		0.2582	0.5475	0.5603	0.5639	0.7138
Physical activity		0.1491	0.4778	0.6004	0.5639	0.6741
Sleep		0.9354	0.4837	0.9215	0.5639	0.6849
Stress		0.1091	0.6647	0.4481	0.5639	0.6586
All		0.1336	0.5320	0.5753	0.5639	0.6531

a SBERT: sentence-bidirectional encoder representations from transformers.

b BERT: bidirectional encoder representations from transformers.

c LaBSE: language-agnostic bidirectional encoder representations from transformers sentence embedding.

**Figure 4.**
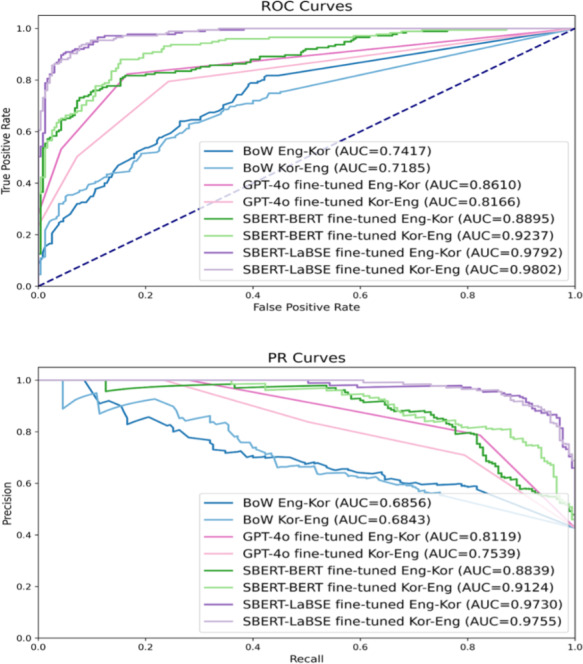
Performance of the cross-language semantic similarity determination. AUC: area under the curve; BERT: bidirectional encoder representations from transformers; BoW: bag-of-words; Eng: English; Kor: Korean; LaBSE: language-agnostic bidirectional encoder representations from transformers sentence embedding; PR: precision-recall; ROC: receiver operating characteristic; SBERT: sentence-bidirectional encoder representations from transformers.

Figure 4 illustrates that SBERT-LaBSE effectively determined semantic similarities between the 2 languages, with slightly better performance in identifying the semantic similarities of English questions relative to the Korean seed questions. The complete results of the cross-language semantic similarity analysis are provided in Multimedia Appendix 4.

## Discussion

### Principal Findings

This study demonstrates the utility of large language models for determining semantic similarities among health questions to facilitate the standardization of survey-based health data. Our results indicate that the fine-tuned SBERT algorithms were significantly more effective than the traditional BoW approach in identifying semantic similarities for both English and Korean questions. Furthermore, the SBERT-LaBSE algorithm demonstrated superior performance particularly for Korean questions, suggesting that it is a more effective method than the SBERT-BERT algorithm, which relies on English translation, for assessing semantic similarity in non-English texts. Notably, the SBERT-LaBSE algorithm outperformed the GPT-4o algorithm, particularly in Korean. Although it is possible that the full potential of the GPT-4o algorithm was not realized, the results clearly show that for the specific task examined in this study, the fine-tuned SBERT algorithms achieved better performance than GPT-4o, with significantly lower computational costs [46].

The SBERT-LaBSE algorithm’s success with Korean questions can be attributed to its structural design and the limitations of language translation. Structurally, LaBSE aligns semantically equivalent words or sentences from different languages into a unified embedding space, preserving semantic consistency across languages. This allows for more accurate semantic similarity assessments. In contrast, the SBERT-BERT algorithm’s lower performance with Korean questions may be due to meaning loss or distortion during translation, which disrupts semantic comparisons between languages [28,29]. Although previous studies have noted that LaBSE may struggle with subtle, sentence-level nuances, limiting its performance in fine-grained similarity tasks [47], our study shows that the SBERT-LaBSE algorithm effectively captured the meanings in both English and Korean sentences, outperforming the SBERT-BERT model. However, this finding should be validated with a larger and more diverse dataset that includes a broader range of syntactic features.

### Limitations

When implemented in the SPURT system with 1835 questions in the comparison space, the SBERT-LaBSE algorithm evaluated the similarity of a new question in just 0.03 seconds. This was achieved on a Naver Cloud Platform server with 8GB RAM and no graphics processing unit [48]. Despite its impressive performance, LaBSE’s 440 million parameters—4 times that of BERT base—make it a resource-intensive option, potentially increasing costs for complex tasks. This resource demand may limit its applicability on resource-constrained devices such as mobile platforms [49]. To address these limitations, future work will explore techniques such as distillation [50] and the use of small language models [51], with the goal of reducing model size while maintaining performance.

This study has some limitations. First, the cutoff values for the similarity scores were not uniformly calibrated across the 5 health lifelog domains, leading to inconsistencies in how similarity scores were interpreted. For example, the SBERT-LaBSE algorithm assigned a similarity score of 0.7 to the dietary question pair “I’ve binge eaten” and “Do you ever overeat?” and identified them as similar. However, the algorithm correctly identified the human living environment questions, that is, “Have you moved in the past 5 years?” and “In the last 5 years, the number of people in this community has?” as dissimilar while assigning the same similarity score of 0.7 to the pair. These inconsistencies may impact the accurate interpretation of similarity scores, highlighting the need for future work to focus on calibrating and normalizing scores across the health lifelog domains to ensure greater consistency. Second, our evaluation was conducted on a small set of English and Korean question pairs. Future studies should explore the feasibility of applying the SBERT-LaBSE algorithm to a broader range of sentence types from diverse domains. Additionally, by incorporating texts from more diverse languages, future research can investigate the algorithm’s potential to overcome language barriers and facilitate semantic interoperability.

### Comparison With Prior Work

Previous methods that relied on metadata tagging [17,18] and ontology-mediated annotation [10,11] were effective in providing structured mappings between concepts, facilitating interoperability. However, they struggled with comparing the meanings of survey questions composed in multiple languages and addressing semantically redundant questions. This study leverages fine-tuned large language models such as SBERT-BERT and SBERT-LaBSE to assess semantic similarity. In particular, the fine-tuned SBERT-LaBSE algorithm demonstrates the potential to enhance semantic interoperability by capturing semantic similarities across multiple languages with high performance.

### Conclusion

This study highlights the potential of large language models in identifying semantic redundancy in survey-based PGHD collections. Specifically, the SBERT-LaBSE algorithm excelled in classifying semantic similarity across diverse question formats in 2 languages. Our findings demonstrate that SBERT-LaBSE outperforms the traditional BERT-based algorithm, the GPT-4o algorithm, and the conventional BoW approach in both languages, highlighting its capacity to improve semantic interoperability of PGHD across language barriers.

## Supplementary material

10.2196/71687 Multimedia Appendix 1 Comparison of algorithms' performance on the semantic textual similarity dataset.

10.2196/71687 Multimedia Appendix 2 The instructions used for GPT-4o semantic similarity evaluation.

10.2196/71687 Multimedia Appendix 3 Example question pairs with the scores from human review and predictions from the 3 algorithms.

10.2196/71687 Multimedia Appendix 4 Performance metrics in the cross-language semantic similarity analysis.

## Abbreviations

BERT: bidirectional encoder representations from transformers
BoW: bag-of-words
CDE: common data elements
LaBSE: language-agnostic bidirectional encoder representations from transformers sentence embedding
NIH: National Institutes of Health
PGHD: person-generated health data
PROMIS: Patient-Reported Outcomes Measurement Information System
SBERT: sentence-bidirectional encoder representations from transformers
SPURT: Standardized Patient-Generated Health Data Utilization Resources and Tools
STS: semantic textual similarity
