# Supplementary material for: Detecting Redundant Health Survey Questions by Using Language-Agnostic Bidirectional Encoder Representations From Transformers Sentence Embedding: Algorithm Development Study
Source: JMIR Med Inform. 2025 Jun 10;13:e71687. doi: 10.2196/71687 (PMC12173092; doi:10.2196/71687)
Supplement: Multimedia Appendix 2 [file medinform-v13-e71687-s002.docx]

**Multimedia Appendix 2.** The instructions used for GPT-4o Semantic Similarity Evaluation

| Prompt via API |
| --- |
| “Evaluate the similarity between the following two sentences on a scale from 1 to 4. Use the following scoring protocol to determine the score: \n\n4 - Minor differences in word choice from the seed question, but takes the same form of response. \n3 - Share the same key topics, though some details may be added, altered, or omitted compared to the seed question. \n2 - The key topics are similar but more specific or general than that of the seed question. \n1 - Does not share the core topic from the seed question or belongs to a completely different health lifelog domain. \n \n Respond with only a single number. \n Sentence 1: {sentence1}\n Sentence 2: {sentence2}” |
