# Supplementary material for: Detecting Redundant Health Survey Questions by Using Language-Agnostic Bidirectional Encoder Representations From Transformers Sentence Embedding: Algorithm Development Study
Source: JMIR Med Inform. 2025 Jun 10;13:e71687. doi: 10.2196/71687 (PMC12173092; doi:10.2196/71687)
Supplement: Multimedia Appendix 3 [file medinform-v13-e71687-s003.docx]

**Multimedia Appendix 3.** Example question pairs with the scores from human review and predictions from the three algorithms.

**Table S1.** Question pairs between English and English in the Physical Activity domain.

S: Similar, D: Dissimilar

| **Seed question:** In the past month, have you ever had chest pain when you were not performing any physical activity? | | | | | | |
| --- | --- | --- | --- | --- | --- | --- |
| Question | score | BoW | SBERT-BERT | | SBERT-LaBSE | |
|  |  |  | zero-shot | fine-tuned | zero-shot | fine-tuned |
| In the past month, have you had chest pain when you were not doing physical activity? | 4 | S | S | S | S | S |
| In the last month, have you experienced chest pain even when you weren't exercising? | 4 | S | S | S | S | S |
| Do you feel pain in your chest when you do physical activity? | 3 | S | D | S | S | S |
| Do you feel pain in your chest when you perform physical activity? | 3 | S | D | S | S | S |
| Have you ever felt pain in your chest when you exercise? | 3 | S | S | S | S | S |
| Do you have chest pain when you exercise? | 3 | S | D | S | S | S |
| Do you often have chest pain and suffer from it? | 3 | S | D | S | D | S |
| Has your doctor ever said that you have a heart condition and that you should only perform physical activity recommended by a doctor? | 2 | S | D | D | D | D |
| In the past month, how often did you have problems with participating in sports activity or exercise? | 2 | S | S | D | S | D |
| Were you sick last week, or did anything prevent you from doing your normal physical activities? | 2 | D | S | D | S | D |
| During the past 4 weeks, how much did physical health problems limit your usual physical activities (such as walking or climbing stairs)? | 2 | S | S | D | D | D |
| I’m in too much pain to exercise | 2 | D | D | D | D | D |
| Have you done general conditioning exercises in the past 4 weeks? | 1 | D | D | D | S | D |
| Have you done moderate to heavy strength training in the past 4 weeks? | 1 | D | D | D | S | D |
| Have you walked leisurely for exercise or pleasure in the past 4 weeks? | 1 | D | S | D | S | D |
| Have you done yoga or Tai-chi in the past 4 weeks? | 1 | D | D | D | D | D |
| I rarely or never do any physical activities. | 1 | S | D | D | D | D |

**Table S2.** Question pairs between Korean and Korean in the Stress Management domain.

S: Similar, D: Dissimilar

| **Seed question:** 일상적인 것이 아닌 사건들(범죄, 자연재해, 우발사고, 이사 등)로 인한 압박감의 정도는?  What is the degree of pressure from cases that are not everyday events (crime, natural disasters, accident accidents, moving, etc.)? | | | | | | |
| --- | --- | --- | --- | --- | --- | --- |
| Question | score | BoW | SBERT-BERT | | SBERT-LaBSE | |
|  |  |  | zero-shot | fine-tuned | zero-shot | fine-tuned |
| 최근 한 달 동안 우발사고로 인해 받은 스트레스의 정도는 어느 정도였나요?  (What was the degree of stress that was caused by contingent accidents in recent months?) | 4 | S | S | S | S | S |
| 최근 한 달 동안 범죄와 같은 일상적이지 않은 사건으로 인해 느낀 스트레스의 정도는 어느 정도였나요?  (What was the degree of stress that you felt because of a routine incident like crime in recent months?) | 4 | D | S | S | S | S |
| 지난 한 달 동안 일상적이지 않은 사건들로 인해 스트레스를 자주 받았었나요?  (Have you been stressed often for the last month?) | 3 | D | D | D | S | S |
| 지난 한 달 동안 예상치 못한 일로 인해 스트레스를 받은 적이 얼마나 자주 있었나요?  (How often have you been stressed for unexpected things over the past month?) | 3 | D | D | S | S | S |
| 지난 한 달 동안 자신의 범죄나 사고로 인해 스트레스를 받은 적이 얼마나 자주 있었나요?  (How often have you been stressed by your crimes or accidents over the past month?) | 3 | S | D | S | D | S |
| 지난 한 달 동안 일상적이지 않은 일로 인해 화가 난 적이 얼마나 자주 있었나요?  (How often have you been angry for the last month?) | 3 | D | D | D | D | S |
| 지난 한 달 동안 갑작스러운 사건들로 인해 스트레스를 받은 적이 얼마나 자주 있었나요?  (How often have you been stressed by sudden events over the past month?) | 3 | D | D | S | S | S |
| (자신 혹은 타인의) 병이나 상해에 의한 압박감의 정도는?  (What is the degree of pressure caused by illness or injury?) | 2 | D | S | S | D | D |
| 대인관계의 변화(사망, 출생, 결혼, 이혼 등)로 인한 압박감의 정도는?  (What is the degree of pressure from the change of interpersonal relationships (death, birth, marriage, divorce, etc.)?) | 2 | D | S | S | S | D |
| (자신이 원했든지 아니든지 간에)일, 직업 및 학교와 관계된 압박감의 정도는?  (What is the degree of pressure related to work, job and school?) | 2 | D | D | D | D | D |
| 지난 일주간 전반적으로 느끼는 압박감의 정도는?  (What is the degree of pressure you feel overall in the past week?) | 2 | D | D | D | D | D |
| 일상생활 중에 일어나는 사소한 변화(동회, 우체국 등을 찾아가는 일 등) 때문에 생기는 압박감의 정도는(만약 일상생활중 변화가 없다면 그것(권태)때문에 생기는 압박감의 정도를 표기하십시요)?  (The degree of pressure from the minor changes that occur in everyday life (such as a club, a post office, etc.) is the degree of pressure (if there is no change in everyday life, indicate the degree of pressure from it (boredom))?) | 2 | S | S | S | S | D |
| 내 업무는 창의력을 필요로 한다.  (My work requires creativity.) | 1 | D | D | D | D | D |
| 내 업무를 수행하기 위해서는 높은 수준의 기술이나 지식이 필요하다.  (In order to perform my work, high levels of technology or knowledge are required.) | 1 | D | S | D | D | D |
| 권위자(윗사람, 직장상관 등)와 맞서게 되었다.  (He was confronted with the authority (superior, workplace, etc.).) | 1 | D | D | D | D | D |
| 지난 한 달간 어느 정도로 경험했는지 오래 생각하시지 말고 최대한 빨리 응답해주세요. 2. 다른 사람으로부터 비난받거나 심판받는다고 느껴진다.  (Don't think about how long you have experienced in the past month, but as soon as possible.2. It feels like being criticized or judged by others.) | 1 | D | D | D | D | D |
| 제시간에 맞추기 위해 서둘러야 했다. (I had to hurry to match the time.) | 4 | S | S | S | S | S |

**Table S3.** Question pairs between English and Korean in the Living Environment domain.

S: Similar, D: Dissimilar

| **Seed question:** Do you have any complaints about your housing and its environment from those mentioned below: noise from neighbors and from outside | | | | | | |
| --- | --- | --- | --- | --- | --- | --- |
| Question | score | BoW | SBERT-BERT | | SBERT-LaBSE | |
|  |  |  | zero-shot | fine-tuned | zero-shot | fine-tuned |
| 귀 댁이 현재 살고 계신 주거환경에 어느 정도 만족하십니까? 가장 많이 이용하는 시설이나 장소를 기준으로 응답해주십시오. 11) 자동차 경적, 집주변의 소음 정도  (How satisfied you are in your living environment? Please respond based on the most common facilities or places. 11) Car horn, degree of noise around the house) | 4 | S | D | S | S | S |
| 귀 댁이 현재 거주하는 주택의상태에 대해 평가해주십시오. 시설이 없는 경우는 불량으로 표기해주십시오. 6-1) 주택 외부 소음(차량 경적, 공사장 소음 등)  (Please evaluate the status of your home. If there is no facility, please mark it in bad. 6-1) External noise outside housing (vehicle horn, construction noise, etc.)) | 4 | S | S | S | S | S |
| 쾌적한 주거환경 조성을 위해 어떠한 부분이 개선이 필요하다고 생각하십니까? 2) 외부 소음 (Do you think that needs improvement to create a pleasant residential environment? 2) External noise) | 3 | S | S | S | D | S |
| 현재 거주하는 주택의 외부 소음 때문에 생활하기가 어렵다.  (It is difficult to live because of the external noise of the current house.) | 3 | S | S | S | S | S |
| 현재 거주하는 주택은 외부 소음이 거의 없는 편이다.  (Currently, houses living in the current houses are rarely external noise.) | 3 | S | D | D | D | S |
| 현재 거주하는 주택은 외부/내부 소음이 심한 편이다.  (Currently, houses that live in the current and internal noise are severe.) | 3 | S | D | S | S | S |
| 쾌적한 주거환경 조성을 위해 외부 소음 관리가 추가로 더 필요하다.  (External noise management is needed to create a pleasant residential environment.) | 3 | S | S | S | D | S |
| 현재 거주공간에 대한 질문입니다. 아래 항목에 대해 느끼시는 정도를 해당 번호에 표시하여 주십시오. 6) 주택 실내가 좁다/넓다  (This is a question about the current living space. Please display the degree of feeling about the items below. 6) The room is narrow/wide) | 2 | D | D | D | D | D |
| 현재 거주공간에 대한 질문입니다. 아래 항목에 대해 느끼시는 정도를 해당 번호에 표시하여 주십시오. 3) 전반적인 환경이 어둡다/밝다  (This is a question about the current living space. Please display the degree of feeling about the items below. 3) The overall environment is dark/bright) | 2 | S | S | D | S | D |
| 현재 거주공간에 대한 질문입니다. 아래 항목에 대해 느끼시는 정도를 해당 번호에 표시하여 주십시오. 9) 주택 내 여름철 덥다/시원하다  (This is a question about the current living space. Please display the degree of feeling about the items below. 9) Hot summer in the house/cool) | 2 | D | D | D | S | D |
| 현재 거주공간에 대한 질문입니다. 아래 항목에 대해 느끼시는 정도를 해당 번호에 표시하여 주십시오. 8) 주택 내 겨울철 춥다/따뜻하다  (This is a question about the current living space. Please display the degree of feeling about the items below. 8) It is cold in winter in the house/warm) | 2 | D | D | D | D | D |
| 귀 댁이 현재 살고 계신 주거환경에 어느 정도 만족하십니까? 가장 많이 이용하는 시설이나 장소를 기준으로 응답해주십시오. 10) 치안 및 범죄 등 방범 상태  (How satisfied you are in your living environment? Please respond based on the most common facilities or places. 10) security and crime status such as security and crime) | 2 | D | D | D | D | D |
| 귀하는 향후 10년 이후에도 현재 자치구에서 거주하고 싶으십니까?  (Do you want to live in autonomous districts after the next 10 years?) | 1 | D | D | D | D | D |
| 귀 댁의 거주 위치는 어디에 해당됩니까? (Where is the location of your home?) | 1 | D | D | D | D | D |
| 이전 거주 주택 유형이 어떻게 되십니까? (What is the type of housing in the previous residence?) | 1 | S | D | D | D | D |
| 귀 댁의 점유형태는 어디에 해당됩니까? (Where is the occupancy of your house?) | 1 | D | D | D | D | D |
| 귀하는 현재 자치구에서 사신 지 몇 년이 되셨습니까? 총 거주기간을 말씀해 주십시오.  (How many years have you been living in autonomous districts? Please tell us the total residence period.) | 1 | D | D | D | D | D |

**Table S4.** Question pairs between Korean and English in the Diet domain.

S: Similar, D: Dissimilar

| **Seed question:** 억제할 수 없이 폭식을 한 적이 있다. (I have been binge eating without suppressing) | | | | | | |
| --- | --- | --- | --- | --- | --- | --- |
| Question | score | BoW | SBERT-BERT | | SBERT-LaBSE | |
|  |  |  | zero-shot | fine-tuned | zero-shot | fine-tuned |
| Have you ever experienced a loss of control while eating an unusually large amount of food, feeling unable to stop or manage what you were eating? | 4 | S | S | S | D | S |
| Have you had instances where you ate significantly more food than usual and felt you couldn't control your eating habits? | 4 | D | D | S | D | S |
| How many TIMES per week on average over the past 3 MONTHS have you eaten an unusually large amount of food and experienced a loss of control? | 3 | D | D | D | D | S |
| How many DAYS per week on average over the past 6 MONTHS have you eaten an unusually large amount of food and experienced a loss of control? | 3 | D | D | D | D | S |
| Feel very upset about your uncontrollable overeating or resulting weight gain? | 3 | D | D | D | D | S |
| How often do you find yourself engaging in binge eating behaviors? | 3 | S | S | S | D | S |
| Do you feel a loss of control while binge eating? | 3 | S | D | D | D | S |
| Eat large amounts of food when you didn't feel physically hungry? | 2 | D | S | D | D | D |
| During the past 6 months have there been times when you felt you have eaten what other people would regard as an unusually large amount of food (e.g. a quart of ice cream) given the circumstances? | 2 | D | D | D | D | D |
| Eat until you felt uncomfortably full? | 2 | D | D | D | D | D |
| Eating disorder | 2 | S | D | D | D | D |
| How often do you find yourself eating excessively, regardless of hunger cues? | 2 | S | S | S | D | D |
| I fear I may start choking when I eat food. | 1 | D | D | D | D | D |
| Has food intake declined over the past 3 months due to loss of appetite, digestive problems, chewing or swallowing difficulties? | 1 | D | D | D | D | D |
| Overall, when you think about the foods you ate over the past 12 months, would you say your diet was high, medium, or low in fat? | 1 | D | D | D | D | D |
| I don't enjoy eating anymore. | 1 | S | D | D | D | D |
| Hyperorality/food fads: Has s/he been drinking or eating excessively anything in sight, or developing food fads, a sweet tooth, eating bananas or cookies excessively, or even putting objects in his/her mouth, or has s/he always had a large appetite and the eating habits have not changed? Has s/he lost table manners? | 1 | S | S | D | D | D |
