# Supplementary material for: Detecting Redundant Health Survey Questions by Using Language-Agnostic Bidirectional Encoder Representations From Transformers Sentence Embedding: Algorithm Development Study
Source: JMIR Med Inform. 2025 Jun 10;13:e71687. doi: 10.2196/71687 (PMC12173092; doi:10.2196/71687)
Supplement: Multimedia Appendix 4 [file medinform-v13-e71687-s004.docx]

**Multimedia Appendix 4.** Performance metrics in the cross-language semantic similarity analysis

**Table S1**. Performance metrics on question pairs with English and Korean seed questions.

| Performance  Metrics | English-Korean question pairs (N=410) | | | | |  | Korean-English question pairs (N=410) | | | | |
| --- | --- | --- | --- | --- | --- | --- | --- | --- | --- | --- | --- |
|  | BoW | GPT-4o  pre-trained | GPT-4o  fine-tuned | SBERT  with fine-tuned | |  | BoW | GPT-4o  pre-trained | GPT-4o  fine-tuned | SBERT  with fine-tuned | |
|  |  |  |  | BERT-base | LaBSE |  |  |  |  | BERT-base | LaBSE |
| Accuracy | 0.6780 | 0.7049 | 0.8439 | 0.8429 | 0.9317 |  | 0.6463 | 0.6976 | 0.8488 | 0.8488 | 0.9268 |
| Precision | 0.5885 | 0.7143 | 0.9512 | 0.8358 | 0.9298 |  | 0.5647 | 0.7383 | 0.9520 | 0.7703 | 0.8962 |
| Recall | 0.8171 | 0.5143 | 0.6686 | 0.7886 | 0.9086 |  | 0.7486 | 0.4514 | 0.6800 | 0.9200 | 0.9371 |
| F1 | 0.6842 | 0.5980 | 0.7852 | 0.8110 | 0.9191 |  | 0.6437 | 0.5603 | 0.7933 | 0.8385 | 0.9162 |
| ROC AUC | 0.7419 | 0.7405 | 0.8868 | 0.8905 | 0.9792 |  | 0.7183 | 0.7183 | 0.8924 | 0.9318 | 0.9802 |
| PR AUC | 0.6857 | 0.6234 | 0.8329 | 0.8911 | 0.9730 |  | 0.6841 | 0.6099 | 0.8385 | 0.9144 | 0.9755 |

**Table S2**. Performance Metrics of the SBERT based algorithms with the fine-tuned BERT and LaBSE models by the health lifelog domains

DL: Dietary Lifestyle, HLE: Human Living Environment, PA: Physical Activity

|  | Performance  Metrics | English-Korean question pairs (N=410) | | | | | |  | Korean-Engish question pairs (N=410) | | | | | |
| --- | --- | --- | --- | --- | --- | --- | --- | --- | --- | --- | --- | --- | --- | --- |
|  |  | DL  (N=80) | HLE  (N=80) | PA  (N=80) | Sleep  (N=85) | Stress  (N=85) | All |  | DL  (N=80) | HLE  (N=80) | PA  (N=80) | Sleep  (N=85) | Stress  (N=85) | All |
| BoW | Accuracy | 0.6875 | 0.8375 | 0.6875 | 0.6118 | 0.7176 | 0.6780 |  | 0.6250 | 0.7250 | 0.7750 | 0.4118 | 0.7294 | 0.6463 |
|  | Precision | 0.5962 | 0.7750 | 0.6190 | 0.5208 | 0.6341 | 0.5885 |  | 0.5490 | 0.6444 | 0.7931 | 0.4118 | 0.6667 | 0.5647 |
|  | Recall | 0.8857 | 0.8857 | 0.7429 | 0.7143 | 0.7429 | 0.8171 |  | 0.8000 | 0.8286 | 0.6571 | 1.0000 | 0.6857 | 0.7486 |
|  | F1 | 0.7126 | 0.8267 | 0.6753 | 0.6024 | 0.6842 | 0.6842 |  | 0.6512 | 0.7250 | 0.7188 | 0.5833 | 0.6761 | 0.6437 |
|  | ROC AUC | 0.7394 | 0.8984 | 0.7048 | 0.6769 | 0.7237 | 0.7419 |  | 0.6613 | 0.7683 | 0.7683 | 0.6449 | 0.7263 | 0.7183 |
|  | PR AUC | 0.6744 | 0.8912 | 0.6594 | 0.6503 | 0.6394 | 0.6857 |  | 0.6215 | 0.7281 | 0.7445 | 0.6697 | 0.6919 | 0.6841 |
| GPT-4o Fine-tuned | Accuracy | 0.8875 | 0.9375 | 0.8375 | 0.8471 | 0.8706 | 0.8366 |  | 0.8875 | 0.9750 | 0.8625 | 0.9412 | 0.9529 | 0.7756 |
|  | Precision | 0.9643 | 1.0000 | 0.8929 | 0.9231 | 0.9615 | 0.8068 |  | 0.8611 | 1.0000 | 0.9286 | 0.9412 | 0.9697 | 0.7085 |
|  | Recall | 0.7714 | 0.8571 | 0.7143 | 0.6857 | 0.7143 | 0.8114 |  | 0.8857 | 0.9429 | 0.7429 | 0.9143 | 0.9143 | 0.8057 |
|  | F1 | 0.8571 | 0.9231 | 0.7937 | 0.7869 | 0.8197 | 0.8091 |  | 0.8732 | 0.9706 | 0.8254 | 0.9275 | 0.9412 | 0.7540 |
|  | ROC AUC | 0.9283 | 0.9429 | 0.8873 | 0.8763 | 0.9291 | 0.8698 |  | 0.9225 | 0.9679 | 0.8946 | 0.9617 | 0.9671 | 0.8290 |
|  | PR AUC | 0.8898 | 0.9376 | 0.8186 | 0.8268 | 0.8766 | 0.8325 |  | 0.8460 | 0.9710 | 0.8401 | 0.9214 | 0.9395 | 0.7716 |
| SBERT with  Fine-tuned  BERT-base | Accuracy | 0.8700 | 0.9025 | 0.7925 | 0.8141 | 0.9176 | 0.8429 |  | 0.8500 | 0.8875 | 0.8625 | 0.8706 | 0.9647 | 0.8488 |
|  | Precision | 0.8155 | 0.8956 | 0.8151 | 0.8287 | 0.9525 | 0.8358 |  | 0.7949 | 0.9063 | 0.7857 | 0.8529 | 0.9706 | 0.7703 |
|  | Recall | 0.9143 | 0.8800 | 0.6971 | 0.6914 | 0.8457 | 0.7886 |  | 0.8857 | 0.8286 | 0.9429 | 0.8286 | 0.9429 | 0.9200 |
|  | F1 | 0.8608 | 0.8876 | 0.7453 | 0.7537 | 0.8947 | 0.8110 |  | 0.8378 | 0.8657 | 0.8571 | 0.8406 | 0.9565 | 0.8385 |
|  | ROC AUC | 0.9236 | 0.9468 | 0.8319 | 0.8398 | 0.9322 | 0.8905 |  | 0.9067 | 0.9473 | 0.9295 | 0.9251 | 0.9931 | 0.9318 |
|  | PR AUC | 0.9125 | 0.9411 | 0.8491 | 0.8352 | 0.9382 | 0.8911 |  | 0.8802 | 0.9256 | 0.9194 | 0.9218 | 0.9911 | 0.9144 |
| SBERT with  Fine-tuned  LaBSE | Accuracy | 0.9000 | 1.0000 | 0.9625 | 0.9294 | 0.9765 | 0.9317 |  | 0.9375 | 0.9875 | 0.9875 | 0.8824 | 0.9765 | 0.9268 |
|  | Precision | 0.8857 | 1.0000 | 0.9211 | 0.8718 | 0.9714 | 0.9298 |  | 0.9412 | 0.9722 | 1.0000 | 0.8049 | 1.0000 | 0.8962 |
|  | Recall | 0.8857 | 1.0000 | 1.0000 | 0.9714 | 0.9714 | 0.9086 |  | 0.9143 | 1.0000 | 0.9714 | 0.9429 | 0.9429 | 0.9371 |
|  | F1 | 0.8857 | 1.0000 | 0.9589 | 0.9189 | 0.9714 | 0.9191 |  | 0.9275 | 0.9859 | 0.9855 | 0.8684 | 0.9706 | 0.9162 |
|  | ROC AUC | 0.9486 | 1.0000 | 0.9924 | 0.9703 | 0.9806 | 0.9792 |  | 0.9778 | 0.9968 | 0.9975 | 0.9543 | 0.9954 | 0.9802 |
|  | PR AUC | 0.9311 | 1.0000 | 0.9900 | 0.9554 | 0.9708 | 0.9730 |  | 0.9744 | 0.9958 | 0.9971 | 0.9387 | 0.9942 | 0.9755 |
